# Targeting ACLY Attenuates Tumor Growth and Acquired Cisplatin Resistance in Ovarian Cancer by Inhibiting the PI3K–AKT Pathway and Activating the AMPK–ROS Pathway

**DOI:** 10.3389/fonc.2021.642229

**Published:** 2021-03-17

**Authors:** Xuan Wei, Juanjuan Shi, Qianhan Lin, Xiaoxue Ma, Yingxin Pang, Hongluan Mao, Rui Li, Wei Lu, Yu Wang, Peishu Liu

**Affiliations:** ^1^Department of Obstetrics and Gynecology, Qilu Hospital of Shandong University, Jinan, China; ^2^Key Laboratory of Gynecology Oncology of Shandong Province, Qilu Hospital of Shandong University, Jinan, China; ^3^Shandong Engineering Laboratory for Urogynecology, Qilu Hospital of Shandong University, Jinan, China; ^4^Department of Gynecology and Obstetrics, Affiliated Tengzhou Center People's Hospital of Jining Medical University, Tengzhou, China

**Keywords:** ACLY, ovarian cancer, cisplatin resistance, PI3K-AKT pathway, AMPK-ROS pathway

## Abstract

**Background:** Ovarian cancer is the most lethal female genital malignancy. Although cisplatin is the first-line chemotherapy to treat ovarian cancer patients along with debulking surgeries, its efficacy is limited due to the high incidence of cisplatin resistance. ATP citrate lyase (ACLY) has been shown to be a key metabolic enzyme and is associated with poor prognosis in various cancers, including ovarian cancer. Nevertheless, no studies have probed the mechanistic relationship between ACLY and cisplatin resistance.

**Methods:** Survival analysis was mainly carried out online. Bioinformatic analysis was performed in R/R studio. Proliferative activity was measured by MTT and colony formation assays. Cell cycle and apoptosis analysis were performed by flow cytometry. The acquired-cisplatin-resistant cell line A2780/CDDP was generated by exposing A2780 to cisplatin at gradually elevated concentrations. MTT assay was used to calculate IC_50_ values of cisplatin. A xenograft tumor assay was used test cell proliferation *in vivo*.

**Results:** Higher expression of ACLY was found in ovarian cancer tissue and related to poor prognosis. Knockdown of ACLY in A2780, SKOV3, and HEY cells inhibited cell proliferation, caused cell-cycle arrest by modulating the P16–CDK4–CCND1 pathway, and induced apoptosis probably by inhibiting p-AKT activity. Bioinformatic analysis of the GSE15709 dataset revealed upregulation of ACLY and activation of PI3K–AKT pathway in cells with acquired cisplatin resistance, in line with observations on A2780/CDDP cells that we generated. Knockdown of ACLY alleviated cisplatin resistance, and works synergistically with cisplatin treatment to induce apoptosis in A2780/CDDP cells by inhibiting the PI3K–AKT pathway and activating AMPK–ROS pathway. The ACLY-specific inhibitor SB-204990 showed the same effect. In A2780/CDDP cells, AKT overexpression could attenuate cisplatin re-sensitization caused by ACLY knockdown.

**Conclusions:** Knockdown of ACLY attenuated cisplatin resistance by inhibiting the PI3K–AKT pathway and activating the AMPK–ROS pathway. These findings suggest that a combination of ACLY inhibition and cisplatin might be an effective strategy for overcoming cisplatin resistance in ovarian cancer.

## Introduction

Ovarian cancer is one of the most lethal tumors worldwide. At the time of diagnosis, the disease is in most cases asymptomatic, and tumors have already spread to other pelvic or extra-pelvic organs. Among females with malignant tumors, ovarian cancer has an incidence rate of only 2.5%, yet it accounts for 5% of all deaths (1). The standard therapy is debulking surgery with platinum-based chemotherapy (2). Although 60–90% of patients with ovarian cancer respond well to first-line platinum-based chemotherapy (3), the median progression-free survival of patients with advanced disease is about 18 months, as most recurrences are platinum-sensitive. Typically, for platinum-sensitive patients, a platinum-based chemotherapy strategy is continued until the cancer develops resistance (4). Many factors account for acquired platinum resistance, including reduced accumulation of the drug (5), increased levels of glutathione (6) and metallothionein (7), and enhanced DNA repair (8, 9). Therefore, ways to prevent acquired platinum resistance and maintain a good response to platinum are urgently required.

ATP citrate lyase (ACLY) is a tetramer consisting of four identical subunits, activated by phosphorylation of the catalytic His760 residue on each N-terminal subunit (10). In the cell, it is located in the cytoplasm, mitochondria and nucleus (11). ACLY catalyzes the synthesis of acetyl-CoA and oxaloacetate (OAA) from citrate and coenzyme A (9). Acetyl-CoA participates in *de novo* synthesis and in the transcription of certain proteins, and catalyzes the acetylation of proteins, especially histones. OAA is a substrate for aspartate production, which is required for nucleotide and polyamine synthesis, and also sustains the regeneration of NAPDH/H^+^, which in turn participates in redox reactions and biosynthesis (12). ACLY connects glucose metabolism with *de novo* lipid synthesis, in which it acts as a key enzyme. High levels of ACLY expression have been detected in many types of tumors, including non-small-cell lung cancer, colorectal cancer, renal cancer, epithelial ovarian cancer, prostate cancer, breast cancer, bladder cancer, hepatocellular cancer, and glioblastomas (13). Targeting ACLY would appear to be novel strategy for tumor therapy.

Our research team has previously investigated ACLY as a prognostic factor of ovarian cancer, and has demonstrated that inhibiting ACLY suppresses the proliferation of ovarian cancer cells (14). In a bioinformatic analysis comparing gene expressing differences in acquired cisplatin-resistant ovarian cancer cells vs. cisplatin-sensitive ones, we found that ACLY and its related pathways were significantly upregulated in cisplatin-resistant cells.

Starting here with a bioinformatic analysis of a GEO dataset, we recognized ACLY to be a key enzyme in regulating acquired platinum resistance. We then investigated the re-sensitization of cells with acquired platinum resistance by ACLY knockdown. Our findings suggest ACLY to be a novel target for maintaining the sensitization of ovarian tumors to platinum.

## Materials and Methods

### Bioinformatic Analysis

Survival analyses of The Cancer Genome Atlas (TCGA) and Gene Expression Omnibus (GEO) datasets from ovarian cancer patients were performed online (http://kmplot.com/analysis/index.php?p=service). GEO datasets (GSE15709) were downloaded (https://www.ncbi.nlm.nih.gov/geo/query/acc.cgi?acc=GSE15709) and analyzed using R/R studio. Differential expressing genes (DEGs) were extracted using the limma R package, with the standard filter of log|FC| ≥1 and *p*-values <0.05. GO and KEGG pathway enrichments were performed by using the clusterProfiler R package (15). Pathway visualization was performed in the Pathview R/Bioconductor package (16). We calculated the rich factor of KEGG enrichment comprehensively with gene numbers and weights using the S4Vectors R package.

### Cell Lines and Culture Conditions

The human epithelial ovarian cancer cell lines A2780, SKOV3, and HEY were purchased from the Cell Bank of the Chinese Academy of Sciences (Shanghai, China). The HEY cells were cultured in Dulbecco's modified Eagle's medium (DMEM) containing 10% fetal bovine serum, and A2780 and SKOV3 cells were cultured in RPMI-1640 medium containing 10% fetal bovine serum. All culture media contained 100 U/ml penicillin and 100 μg/ml streptomycin. Cells were incubated at 37°C in air containing 5% CO_2_.

### Induction of Cisplatin Resistance

A2780/CDDP cells were obtained by exposing A2780 cells to stepwise-increasing concentration of cisplatin. Initial concentration of cisplatin was 2 μM, the cells were exposed to cisplatin for 2 days as a cycle, then cells were allowed for growth recovery in medium without cisplatin between cycles. Cycles were repeated for three times on the same concentration of cisplatin. After the completion of three cycles of same concentration of cisplatin stimulation, the dose was elevated. The procedures were repeated until the final concentration of cisplatin reached 50 μM. Cells were used for subsequent experiment only when they remained the resistance to cisplatin after cultured in medium without cisplatin for at least 6 months.

### Cell Viability and Colony Formation Assays

The MTT assay was used to measure cell proliferative ability. Cells were inoculated into 96-well plates, each well containing 800–1000 cells, and incubated overnight to adhere. Cells were incubated for up to 5 days, and assays performed at intervals of 24 h by adding 10 μl of MTT (5 mg/ml; Sigma–Aldrich, CA, USA) to each well, then the plates were returned to the incubator at 37°C for 4 h. The supernatant was carefully aspirated and 100 μl of DMSO (MP Biomedicals, OH, USA) was added into every well. After the formazan was fully dissolved, plates were placed in the microplate reader (Tecan Group Ltd, Männedorf, Switzerland), and the absorbance at 490 nm was measured.

For colony formation assays, 800 single cells were seeded in six-well plates and mixed with 2 ml of culture medium. After incubation at 37°C for 12 days, cells were fixed with methanol and stained with 0.1% crystal violet (Beyotime, Beijing, China).

### Drug Resistance Assay

MTT assays were used to measure the surviving fractions of cells and cisplatin IC_50_ values. Cells were seeded into 96-well plates at a concentration of 3,000 cells per well, then incubated with different concentrations of cisplatin for 24, 48, or 72 h. Finally, the absorbance was measured by microplate reader as described above.

### Cell Cycle Assay

Cells were harvested and fixed with ethanol when 80% confluency was reached, then stained with propidium iodide according to the manufacturer's protocol (BD, NJ, USA). The treated cells were separated by flow cytometry (FACSCalibur, BD, USA) and analyzed using Modifit LT software.

### Cell Apoptosis Assay

Cells were harvested with trypsin without EDTA, washed three times with phosphate-buffered saline (PBS), and resuspended in annexin binding buffer at a concentration of 1 × 10^6^ per 100 μl. Then, cells were stained with 5 μl annexin V-APC and 10 μl 7-aminoactinomycin D solution (MultiSciences, Hangzhou, China), and incubated at room temperature for 5 min. Finally, cells were separated by flow cytometry (FACSCalibur), and analyzed with Flowjo V10 software.

### Lentivirus Production and Stable Transfections

The sequence of shACLY is: 5′-CCATCATAGCTGAAGGCAT-3′, NC: 5′-TTCTCCGAACGTGTCACGT-3′. The plasmid containing shACLY and the corresponding NC were purchased from Genechem (Shanghai, China). HEK279T cells were used to produce lentivirus. To that end, HEK293T cells were transfected (Lipofectamine 3000) with the shACLY plasmid together with pMD2.G and psPAX2. Ovarian cancer cells were infected with the obtained lentivirus over a period of 12 h. These cells were then cultured with medium containing 2 μg/mL puromycin (Solabio, Beijing, China) to obtain stably transfected cells.

### Transient Transfection

The plasmid containing AKT and the corresponding NC plasmid were purchased from Genechem (Shanghai, China). Lipofectamine 3000 (Invitrogen, USA) was used to transfect cells with these plasmids. Cells were harvested 24–48 h after transfection for the following assays.

### Quantitative Real-Time Transcription-Polymerase Chain Reaction

Cells' Total RNA was extracted using SteadyPure Universal RNA Extraction Kit, concentration and purity were detected using spectrophotometer (Thermo Fisher Scientific Inc., MA, USA). Then the RNA was transcribed into cDNA. PCR reaction was performed on StepOne™ PCR amplifier (Applied Biosystems, USA) with SYBR-green (TAKARA, Japan) in a 10 μl reaction system. β-actin was used as the endogenous control. Primers for human ACLY gene were as follows: forward: 5′-CAGACGGGCAAAGAACTCCT-3′, reverse: 5′-TCAGGAGTGACCCGAGCATA-3′. Relative gene expression levels were normalized to β-actin. Primers for β-actin gene were as follows: forward: 5′-CTC ACC ATG GAT GAT GAT ATCGC-3′, reverse: 5′-AGG AAT CCT TCT GAC CCA TGC-3′.

### Western Blotting Assays

Adherent cells were washed three times with PBS, lysed with RIPA Lysis Buffer (Beyotin, Beijing, China) containing 1% PMSF and 1% NaF, and placed on ice for 30 min. Lysed cells were subjected to centrifugation to obtain a protein-containing supernatant. The concentration of protein was measured by BCA protein assay (reagents from Beyotime, Beijing, China). An appropriate volume of 5× loading buffer was added to the protein to achieve a final concentration of 1× , and followed by heating for 5 min at TEMP 98°C in a metal bath. Samples were loaded onto 10 or 12% SDS-PAGE gels at 30 μg per well, then separated by electrophoresis. Gels were then transferred onto 0.22-μm polyvinylidene fluoride membranes (Merck Millipore, USA). Membranes were blocked in 5% skimmed milk for 1 h, then incubated with primary antibodies overnight at 4°C, washed with 1× Tris-buffered saline containing tween (TBST), then incubated with HRP-linked secondary antibodies at room temperature for 1.5 h or less. HRP Substrate Reagent (Thermo Fisher Scientific Inc., MA, USA) was used to detect bands on membranes under an Image Quant LAS 4000 (GE Healthcare Life Science). GAPDH was detected as the endogenous control.

### Tumor Xenograft Experiments

A2780 and A2780/CDDP cells stably expressing shACLY and the corresponding NC cell lines were used to establish xenografts. Cells were harvested, and 1 × 10^7^ cells were resuspended in 200 μl PBS and injected subcutaneously on either side of the axilla of 4–6-week-old nude female mice. Tumor sizes were measured every other day from day 10 after injection. The mice bearing A2780/CDDP cells received intraperitoneal injections of cisplatin at a concentration of 4 mg/kg body weight (B.W.) on days 7, 14, and 28 (a total of three injections). Mice that received A2780 cells were sacrificed 35 days after grafting, and those in the A2780/CDDP group were sacrificed 28 days after cisplatin injection. Tumor volumes were calculated as *V* = [(length × width^2^)/2].

### Antibodies and Chemical Inhibitors

Cisplatin was purchased from MCE (NJ, USA), and was solved in phosphate-buffered saline (PBS) with the help of ultrasound, reaching the concentration of 3.33 mM, equal dose of PBS in accordance with cisplatin added as 0 μM control group. The antibodies for ACLY, P16 were purchased from Abcam (Cambridge, UK). The antibodies for cleaved poly (ADP-ribose) polymerase (PARP), P53, pan-AKT, phosphorylated AKT (Ser473), phosphorylated AMPK-α, PI3K, and GAPDH were purchased from Cell Signaling Technology (Danvers, MA, USA). SB-204990 was purchased from MCE (NJ, USA).

### Measurement of Intracellular ROS Levels

The intracellular levels of reactive oxygen species (ROS) were measured using a Reactive Oxygen Species Assay Kit (Beyotime, Shanghai, China). Three thousand cells were seeded in each well of a 96-well plate and exposed to 20 μM cisplatin for 24 h. Following treatment, the cells were incubated with 2′,7′-dichlorodihydrofluorescein diacetate at 37°C for 20 min and their emission at 525 nm (488 nm excitation) was measured with a fluorescence microplate reader.

### Statistical Analysis

All experiments were repeated at least three times. GraphPad Prism 8.0.1 (GraphPad Software, USA) was mainly used in the data analysis. Student's *t*-tests and one-way ANOVA analyses were used to determine the statistical differences among the groups. Data are presented as the mean ± SD unless otherwise stated. IC_50_ values of cells are best fit values at the 95% CI. A *p*-value <0.05 was regarded as statistically significant.

## Results

### ACLY Was Upregulated in Ovarian Cancer Tissues and Was Associated With Poor Prognosis

**Figure 1:**
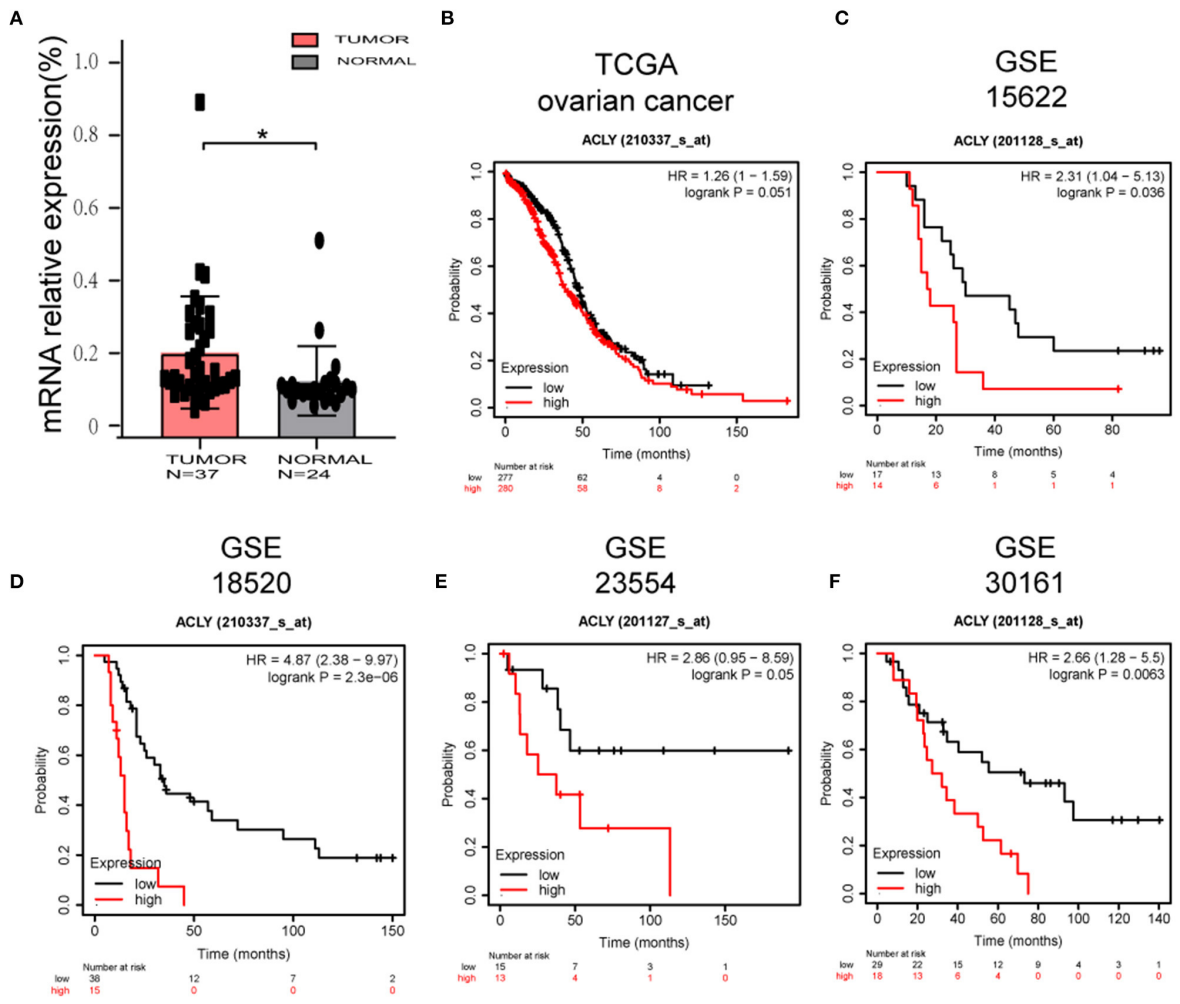
ACLY was upregulated in ovarian cancer tissues, and its expression was associated with poor prognosis. **(A)** The comparison of ACLY expression between high grade serous ovarian cancer tissues (*N* = 47) and fallopian tube epithelium (*N* = 24) of patients from Qilu hospital. Statistical analysis was performed using Student's *t*-test. *P < 0.05. **(B–F)** Overall survival analysis based on ACLY expression (high-expression group vs. low-expression group) of TCGA ovarian cancer cohort, GSE15622 dataset, GSE18520 dataset, GSE23554 dataset, GSE30161 dataset. *P < 0.05 for statistical analysis of the indicated groups.

First, we performed bioinformatic analysis to explore the characteristics of ACLY in ovarian cancer. We measured mRNA levels in tissues obtained from patients at the Qilu Hospital of Shandong University. Tumor tissues (*N* = 47) were diagnosed as high-grade serous ovarian cancer, and normal tissues (*N* = 24) were fallopian tube epithelium from patients that had undergone salpingectomies owing to benign disease. We found that ACLY was upregulated in cancer tissues compared with normal ones, with statistical significance (*p* = 0.0206; Figure 1A). To elucidate the relationship between ACLY expression and prognosis (mainly measured by overall survival), TCGA and GEO datasets were used to perform a bioinformatic analysis. In the five datasets we analyzed, higher expression of ACLY predicted poorer overall survival (Figures 1B–F).

### ACLY Knockdown Inhibited Ovarian Cancer Progression *in vitro* and *in vivo*

**Figure 2:**
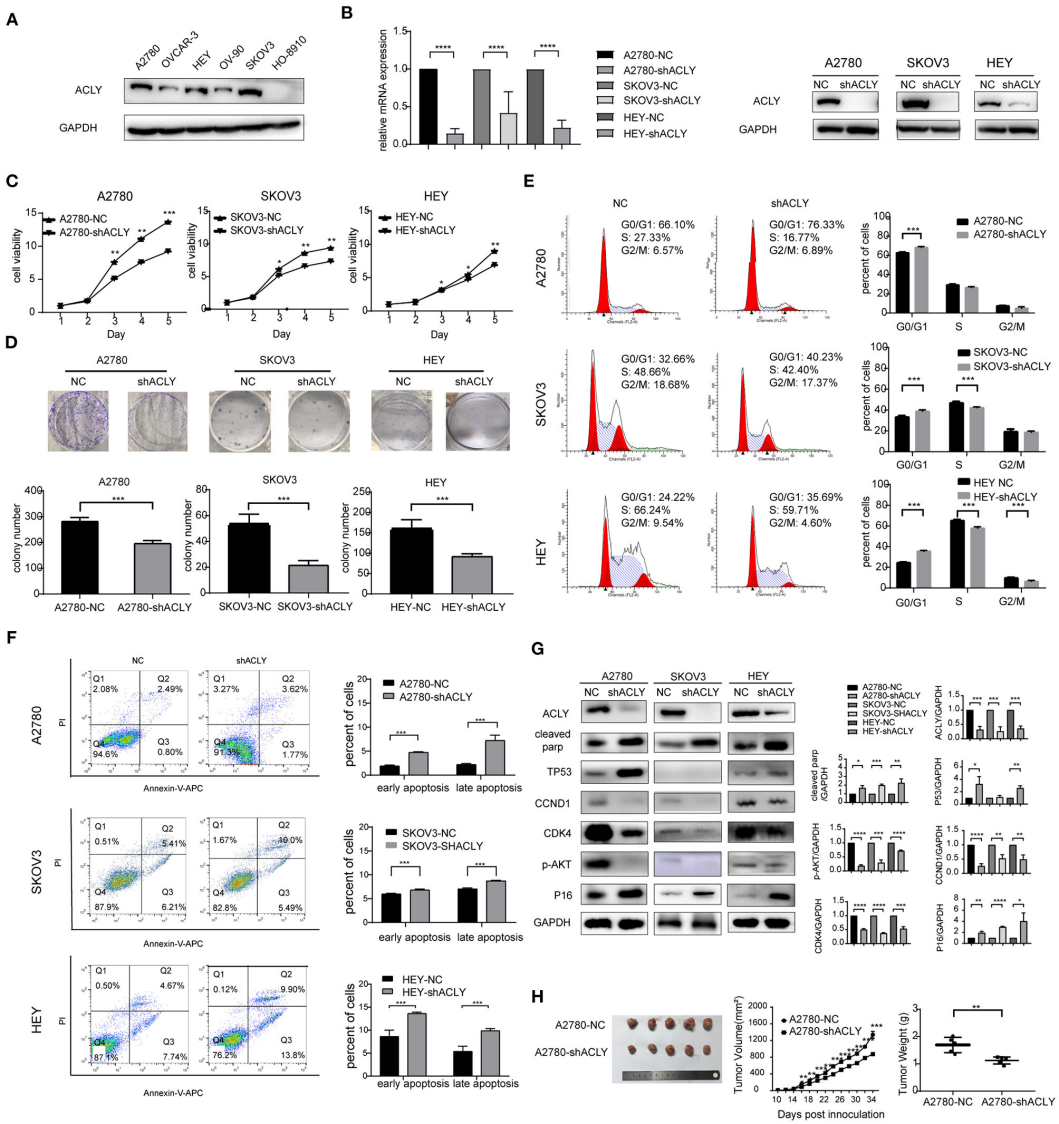
ACLY knockdown inhibited ovarian cancer progression *in vitro* and *in vivo*. **(A)** Western blotting was used to detect the expression of ACLY in several ovarian cancer cell lines. **(B)** PCR and Western blotting was used to confirm the effectiveness of ACLY knockdown by lentivirus in A2780, SKOV3, and HEY cells, the relative mRNA levels were analyzed using Student's *t*-test. **(C)** MTT assays were used to detect the proliferative activity change on the knockdown of ACLY in A2780, SKOV3, and HEY cells, the growth curves were analyzed using one-way ANOVA test. **(D)** Colony formation assays of ACLY knockdown and its corresponding NC cells of A2780, SKOV3, and HEY, colony numbers were counted and analyzed using Student's *t*-test. **(E)** Cell cycle of A2780, SKOV3, and HEY with ACLY knockdown cells and their corresponding NC cells by flowcytometry, the difference of cell cycle distribution between groups was analyzed using student *t*-test. **(F)** Annexin-V apoptosis assays and flowcytometry were used in ACLY knockdown and its corresponding NC cells of A2780, SKOV3, and HEY, quantitive analysis of apoptotic ratio by flowjo V10 software. Statistical analysis was performed using Student's *t*-test. **(G)** Western blotting images, quantitation, and statistical analysis of the western blotting bands in the aforementioned cells to detect the expression of cell cycle checkpoints (CCND1, CDK4), their upstream regulators (P16, P53), marker of apoptosis (cleaved parp), and p-AKT. The bands were quantitated with Image J software, statistical analysis was performed using Student's *t*-test. **(H)** Tumor xenograft formation of A2780-shACLY and A2780-NC cells in nude mice, with each group containing five mice. The difference in volume, growth curves and weights were calculated and presented. The growth curves were analyzed using one-way ANOVA test. NC negative control. The difference of tumor weight between groups was analyzed using Student's *t*-test. All cell experiments were repeated three times at least. *P < 0.05, **P < 0.01, ***P < 0.001, and ****P < 0.0001 for statistical analysis of the indicated groups.

A2780, SKOV3, and HEY cells were selected from six common ovarian cancer cell lines for use in the following experiments, as they expressed relatively higher levels of ACLY (Figure 2A). Lentivirus-transduced cells stably expressed shACLY and NC, PCR and western blotting confirmed the blocking efficacy (Figure 2B). The results of MTT assays revealed that knockdown of ACLY can significantly suppress the ability of ovarian cancer cells to proliferate (Figure 2C), consistent with fewer colonies forming with shACLY cells compared to NC cells (Figure 2D). In a cell-cycle analysis, we found a greater proportion of ACLY-knockdown cells in the G0/G1 phase compared to NC cells, demonstrating that knockdown of ACLY induced arrest of the G1 phase (Figure 2E). The results of apoptosis assays showed knockdown of ACLY caused an increase in apoptosis (Figure 2F). We found the expression of the G1 phase checkpoint markers cyclin D1 (CCND1) and CDK4 were downregulated in ACLY-knockdown cells. P16, as the upstream inhibitor of CDK4, was upregulated in ACLY-knockdown cells (Figure 2G). Compared with SKOV3 (the P53-null cell line), A2780 and HEY cells (expressing wild-type P53) had higher proportions of apoptotic cells and P53 was upregulated upon ACLY knockdown (Figure 2G). We found that cleaved PARP was significantly upregulated in ACLY-knockdown cells (Figure 2G). To further verify the antitumor effect, we used lentivirus-transduced A2780 cells stably transfecting shACLY and NC to construct an *in vivo* xenograft model. The volumes of tumors were significantly smaller in the ACLY-knockdown group than those in the NC group, and the same trend was observed for tumor weight (Figure 2H).

### Bioinformatic Analysis Showed ACLY Is Upregulated in A2780/CDDP Cells

**Figure 3:**
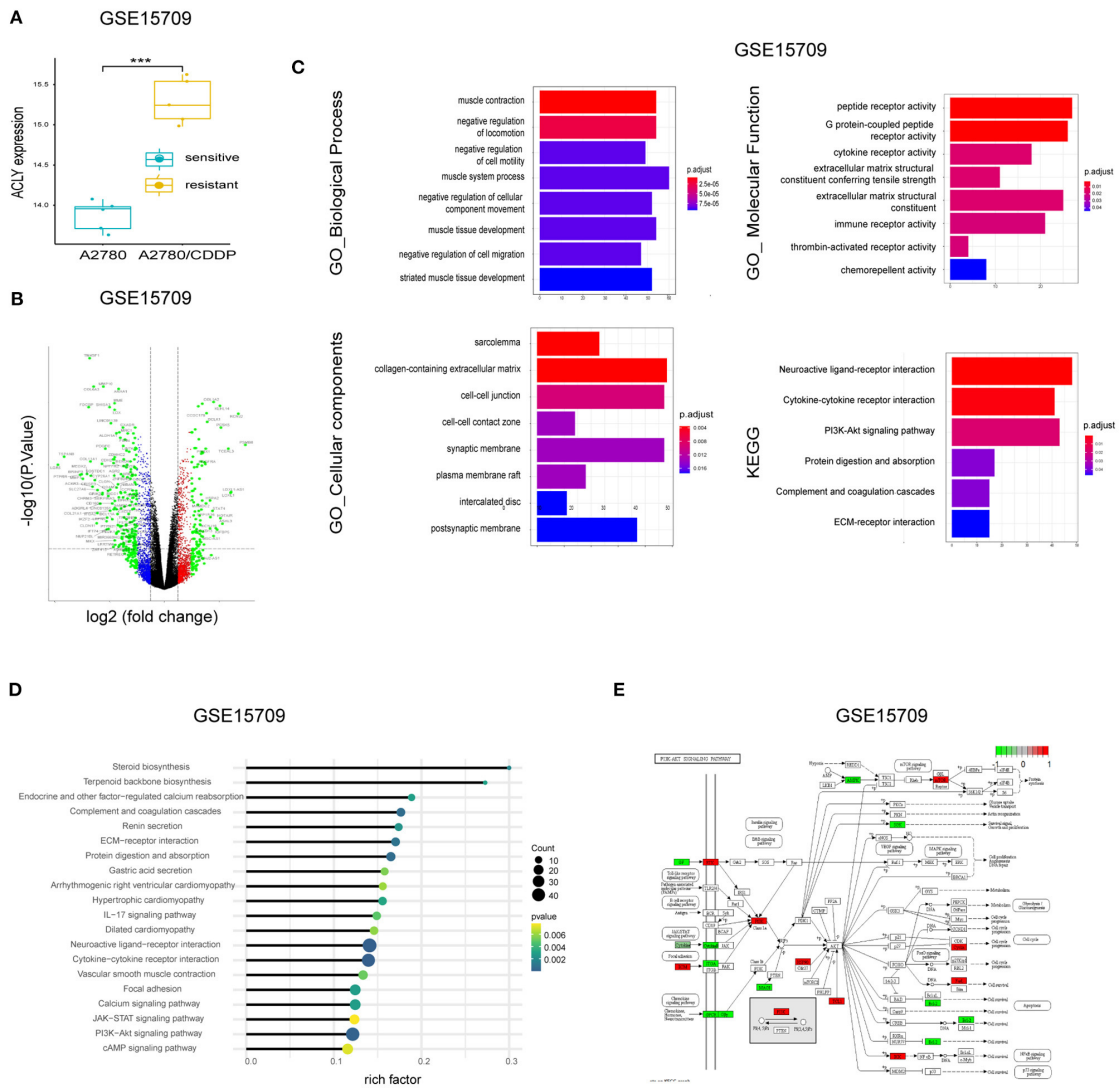
Bioinformatic analysis showed ACLY is upregulated in A2780/CDDP cells. **(A)** ACLY was upregulated in A2780/CDDP cells compared with A2780 cells in GSE15709 dataset. **(B)** The volcano plot on DEGs between A2780 and A2780/CDDP in GSE15709 dataset. **(C)** The GO enrichment and KEGG enrichment of DEGs between A2780/CDDP and A2780, the filtering condition was *p* < 0.05. **(D)** KEGG enrichment of DEGs of GSE15709 calculated and weighted by rich factor. **(E)** Pathway viewer of PI3k/AKT, the red units mean upregulated in A2780/CDDP, and the green units mean downregulated in A2780/CDDP, the darker the color is, the less the *P*-value is. ***P < 0.001 for statistical analysis of the indicated groups.

We screened DEGs between A2780 vs. A2780/CDDP with the GEO dataset GSE15709. We found that ACLY was upregulated in A2780/CDDP cells (Figure 3A). A volcano plot of the DEGs supports this (Figure 3B). We then performed enrichment of DEGs via GO and KEGG pathways (Figure 3C). Our computations showed an obvious activation of the PI3K/AKT pathway in A2780/CDDP cells (Figures 3C–E). In addition, we found that AMPK was downregulated in A2780/CDDP cells (Figure 3E). With the result that knockdown of ACLY downregulated p-AKT in ovarian cancer cells (Figure 2G), we proposed that ACLY knockdown might rescue the cisplatin resistance caused by inhibiting the PI3K–AKT pathway.

### ACLY Knockdown Re-Sensitized A2780/CDDP Cells to Platinum

**Figure 4:**
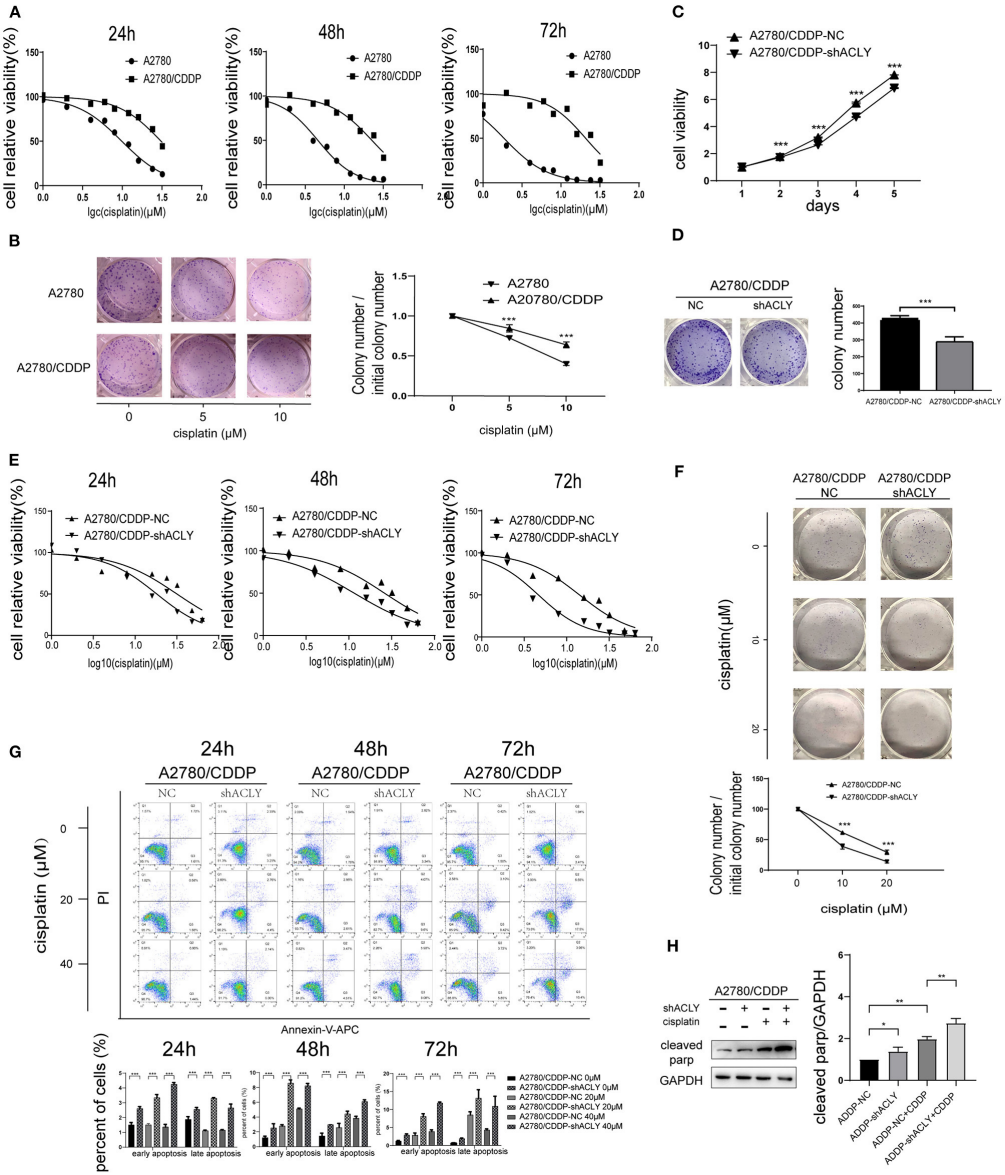
ACLY knockdown re-sensitized A2780/CDDP cell to platinum. **(A)** 24, 48, and 72 h IC_50_ were measured after treatment of cisplatin with concentration gradients. Twenty-four hours IC50 of A2780 and A2780/CDDP were 9.827 (9.316–10.36) and 30.75 (28.91–33.03) μM, respectively, 48 h IC50 were 4.593 (4.355–4.838) and 22.68 (20.88–24.89) μM, respectively, 72 h IC50 were 1.861 (1.749–1.976) and 20.76 (18.39–23.85) μM, respectively. **(B)** Colonies formation assays and the survival ratio of A2780 and A2780/CDDP cells treated with different concentration of cisplatin, colony numbers were counted and analyzed using Student's *t*-test. **(C)** MTT assays were used to measure the proliferative ability of ACLY knockdown A2780/CDDP cells and their corresponding NC cells, the growth curves were analyzed using one-way ANOVA test. **(D)** Colony formation assays were performed and analyzed in ACLY knockdown cells and their corresponding NC cells, colony numbers were counted and analyzed using Student's *t*-test. **(E)**. IC50 of cisplatin of 24, 48, and 72 h after cisplatin gradients added. Twenty-four hours IC50 in A2780/CDDP-NC cells and A2780/CDDP-shACLY cells were 31.71 (26.49–38.15) and 16.64 (14.26–19.30) μM, respectively, 48 h IC50 were 26.07 (22.83–29.74) and 11.55 (10.43–12.76) μM, respectively, 72 h IC50 were 14.16 (12.31–16.19) and 4.648 (4.138–5.227) μM, respectively. **(F)** Colony formation assays were performed in the aforementioned cells under treatment with different concentration of cisplatin, colony numbers were counted and analyzed using Student's *t*-test. **(G)** Apoptosis assays were measured in cells treated with different concentrations and different time of A2780/CDDP-NC and A2780/CDDP-shACLY cells. Quantitive analysis of apoptotic ratio by flowjo V10 software. Statistical analysis was performed using Student's *t*-test. **(H)** cleaved parp was measured and quantified in A2780/CDDP-NC and A2780/CDDP-shACLY cells with or without cisplatin treated, the concentration of cisplatin was 20 μM. The bands were quantitated with Image J software, statistical analysis was performed using Student's *t*-test. All cell experiments were repeated three times at least. *P < 0.05, **P < 0.01, ***P < 0.001, and ****P < 0.0001 for statistical analysis of the indicated groups.

The IC_50_ of cisplatin in A2780/CDDP cells after 48 h treatment was nearly five times that in A2780 cells; after 72 h, the difference was >10-fold (Figure 4A). Colony formation assays also indicated that A2780/CDDP showed greater resistant to cisplatin (Figure 4B). MTT assays showed that ACLY knockdown in A2780/CDDP cells inhibited proliferation and colony formation and lowered the IC_50_ value of cisplatin (Figures 4C–E). A colony formation assay revealed that the survival ratio of cisplatin-treated cells was higher for A2780/CDDP-NC cells than for A2780/CDDP-shACLY cells (Figure 4F). The proportion of apoptotic A2780/CDDP-shACLY cells was significantly higher than that of A2780/CDDP-NC cells, and this difference increased with time and concentration of cisplatin (Figure 4G). The results of western blotting showed that cleaved PARP was upregulated in A2780/CDDP-shACLY cells with or without cisplatin comparing with A2780/CDDP-NC cells (Figure 4H).

### ACLY Knockdown Inhibited PI3K–AKT Pathway and Activated AMPK Pathway

**Figure 5:**
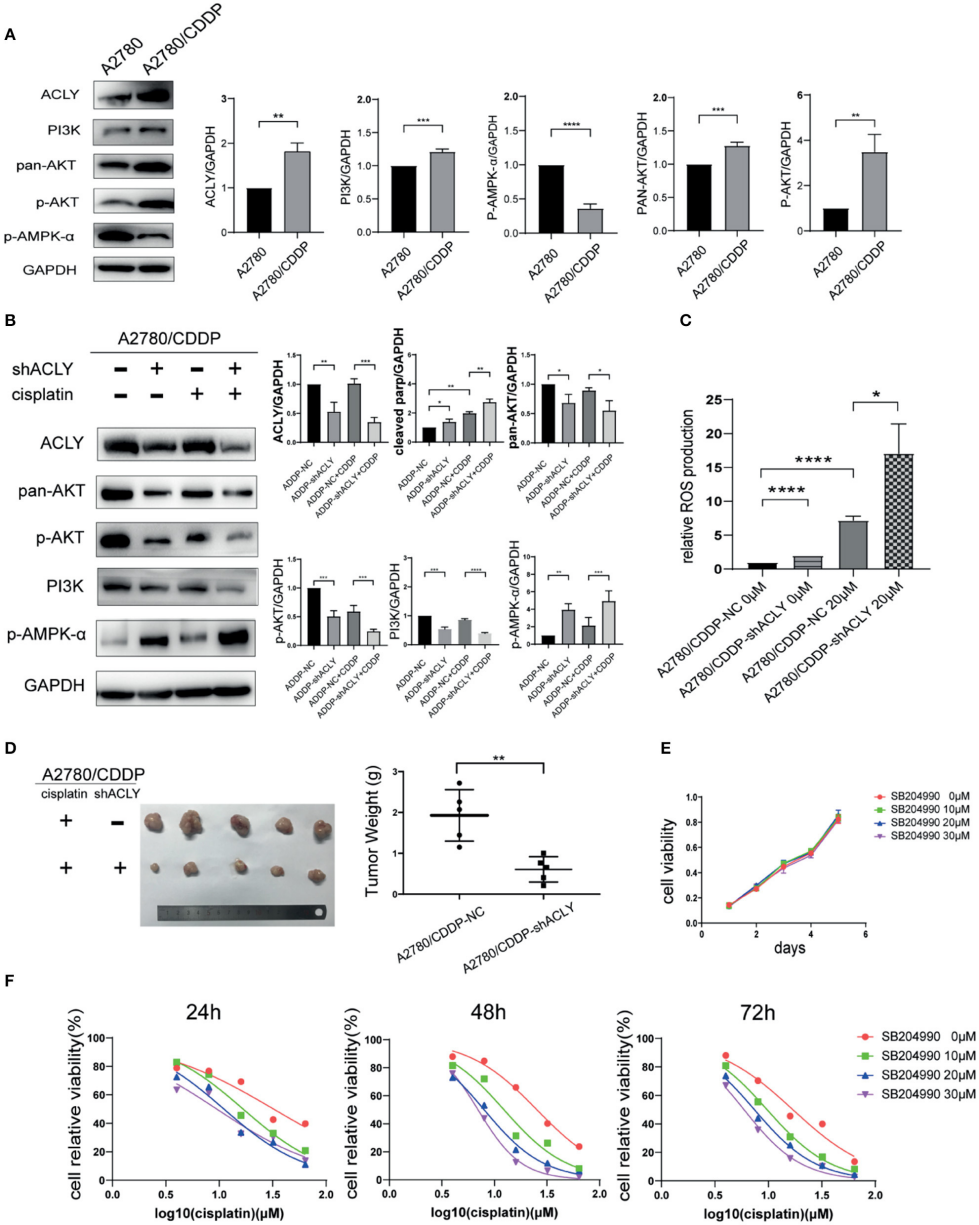
ACLY knockdown inhibited PI3K/AKT pathway and activated AMPK pathway. **(A)** Western blotting was used to detect the differential expression of ACLY, PI3K/AKT pathway and p-AMPK-α in A2780 and A2780/CDDP cells. **(B)** Western blotting on A2780/CDDP-NC and A2780/CDDP-shACLY cells, and them under 20 μM cisplatin treatment for 48 h, the bands were quantified and analyzed. The bands were quantitated with Image J software, statistical analysis was performed using Student's *t*-test. **(C)** ROS production of the aforementioned cells and them under treatment of 20 μM cisplatin for 48 h, statistical analysis was performed using Student's *t*-test. **(D)** Tumor xenograft formation of A2780/CDDP-NC and A2780/CDDP-shACLY cells with treatment of cisplatin, with each group containing five mice. The difference in tumor weights was compared using Student's *t*-test. **(E)** Proliferation of A2780/CDDP cells in respond to different concentration (low-dose, 10–30 μM) of SB-204990, the growth curves were analyzed using one-way ANOVA test. **(F)** 24, 48, and 72 h IC50 of A2780/CDDP cells under treatment of cisplatin combined with different concentration of SB-204990 (from 0 to 30 μM), 24 h IC50 of which were 32.34 (26.71–40.60), 16.75 (15.24–18.43), 11.08 (9.736–12.55), 9.495 (7.759–11.38) μM, respectively; 48 h IC50 of which were 25.37 (23.86–27.00), 12.33 (10.74–14.13), 7.983 (7.487–8.499), 6.979 (6.749–7.215) μM; 72 h IC50 of which were 16.96 (14.89–19.34), 9.727 (9.294–10.18), 7.407 (7.083–7.741), 5.922 (5.601–6.246) μM, respectively. All cell experiments were repeated three times at least. *P < 0.05, **P < 0.01, ***P < 0.001, and ****P < 0.0001 for statistical analysis of the indicated groups.

The results of western blotting show that PI3K, pan-AKT, and p-AKT were upregulated, and p-AMPK-α was downregulated in A2780/CDDP cells (Figure 5A), which is consistent with the bioinformatic analysis of pathway enrichment using the GEO dataset GSE15709. Next, we measured the change of these factors in ACLY-knockdown cells treated with cisplatin. p-AKT was downregulated under cisplatin treatment and the combination with ACLY knockdown further drove a downregulation (Figure 5B). PI3K was downregulated under treatment with cisplatin, but was sharply downregulated in cisplatin-treated ACLY-knockdown cells. ACLY knockdown synergistically activated AMPK-α with cisplatin treatment. Accordance with the activation of AMPK-α, ROS levels increased, especially when ACLY inhibition and cisplatin treatment was combined (Figure 5C). To verify the synergistic antitumor effects of ACLY inhibition and cisplatin treatment *in vivo*, we used A2780/CDDP-shACLY and A2780/CDDP-NC cells to construct tumor models in nude mice. Tumors of A2780/CDDP-shACLY cells were smaller in volume than those of A2780/CDDP-shNC cells, and lighter in weight (Figure 5D).

SB-204990 is a highly potent and specific small-molecule inhibitor of ACLY. With a low dose of SB-204990 (<30 μM), we saw no significant difference in the proliferation of treated cells (Figure 5E). However, SB-204990 showed a synergistic effect with cisplatin in reducing the IC_50_ of cisplatin over time (Figure 5F).

### Overexpression of AKT Increased Resistance of A2780/CDDP-shACLY Cells to Cisplatin

**Figure 6:**
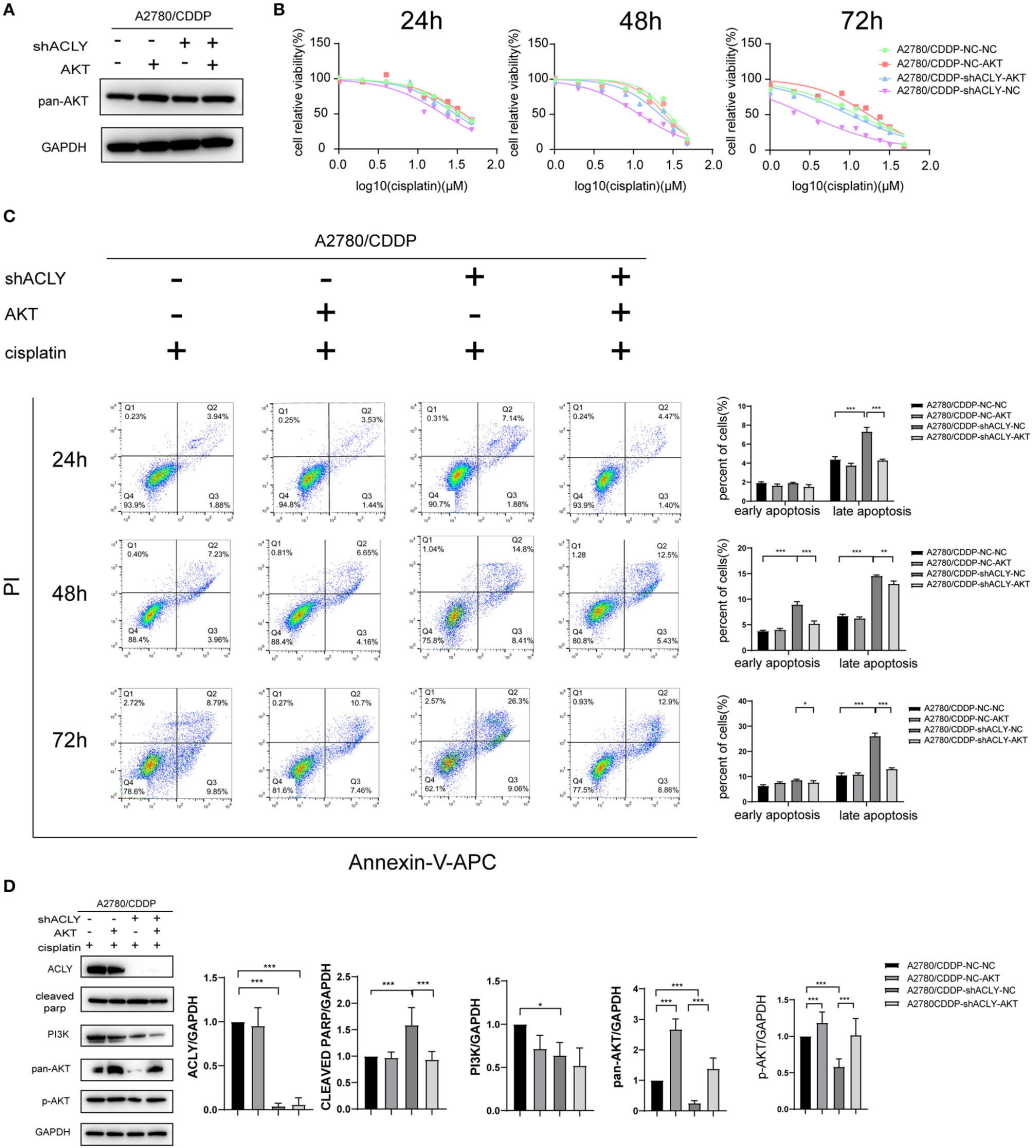
Overexpression of AKT increased resistance of A2780/CDDP-shACLY cells to cisplatin. **(A)** Western blotting was used to confirm the effectiveness of AKT overexpression by plasmid in A2780/CDDP-NC and A2780/CDDP-shACLY cells. **(B)** The 24 h IC50 of cisplatin of A2780/CDDP-NC-NC, A2780/CDDP-NC-AKT, A2780/CDDP-shACLY-NC and A2780/CDDP-shACLY-AKT was 34.33 (33.04–35.74), 38.06 (34.39–42.95), 20.72 (18.83–22.90), and 29.86 (27.38–32.86) μM, respectively; 48 h IC50 of these cells were 27.90 (25.78–30.21), 25.06 (23.07–27.22), 11.86 (11.06–12.70), and 21.56 (18.96–24.44) μM, respectively; 72 h IC50 of these cells were 12.46 (10.88–14.21), 14.26 (12.85–16.74), 2.933 (2.552–3.340), and 9.891 (8.613–11.29) μM, respectively. **(C)** Apoptosis analysis on A2780/CDDP-NC-NC, A2780/CDDP-NC-AKT, A2780/CDDP-shACLY-NC, and A2780/CDDP-shACLY-AKT cells with the treatment of 20 μM cisplatin on different time. Quantitive analysis of apoptotic ratio by flowjo V10 software. Statistical analysis was performed using Student's *t*-test. **(D)** Western blotting was used to detect the expression of PI3K/AKT relative proteins of A2780/CDDP-NC-NC, A2780/CDDP-NC-AKT, A2780/CDDP-shACLY-NC, and A2780/CDDP-shACLY-AKT cells treated with 20 μM cisplatin for 48 h, the bands were quantified and analyzed. The bands were quantitated with Image J software, statistical analysis was performed using Student's *t*-test. All cell experiments were repeated three times at least. *P < 0.05, **P < 0.01, and ***P < 0.001 for statistical analysis of the indicated groups.

We postulated that knockdown of ACLY reduced resistance to cisplatin via downregulation of the PI3K–AKT pathway. Accordingly, we used AKT overexpression to perform a rescue experiment; western blotting verified the overexpression of AKT (Figure 6A). We tested whether overexpression of AKT affected the cisplatin sensitivity of A2780/CDDP-shACLY cells, and found a recovery of the IC_50_ in A2780/CDDP-shACLY-AKT cells compared with A2780/CDDP-shACLY-NC cells (Figure 6B). While, when overexpressed AKT only in A2780/CDDP-NC cells, it did not enhance resistance to cisplatin (Figure 6B). We then wondered whether overexpression might attenuate the higher apoptosis induced by cisplatin treatment of ACLY-knockdown cells. Flow cytometry studies showed overexpression of AKT could partly attenuate the apoptosis induced by ACLY inhibition combined with cisplatin treatment (Figure 6C). PARP cleavage, as detected by western blotting, also showed the same trend in AKT-overexpressing cells (Figure 6D).

## Discussion

Ovarian cancer appears to be the most lethal form of cancer in females. The two main reasons for this are that ovarian cancer is asymptomatic at an early stage, and platinum resistance (intrinsic or acquired). Although initial treatment typically ensures remission, the cancer almost always recurs and acquires resistance to platinum upon further chemotherapy. Finding ways to overcome resistance to platinum is therefore a matter of urgency.

Rapid proliferation is a characteristic of cancer tissues and immortalized cancer cells that necessitates extremely active glucose and lipid metabolism. In particular, an enhanced rate of *de novo* fatty acid synthesis is required for the synthesis of cellular membranes (17). And this can be detected in proliferating tumors (18). In bladder cancer and hepatocellular carcinoma tissues, the expression of several lipogenic enzymes is higher than in normal tissues, and is associated with poor prognosis (19, 20). Qian et al. reported that high ACLY expression correlated with poor prognosis, advanced stages, and lymph node metastasis in gastric cancer (21). Migita et al. showed that ACLY expression was higher in non-small cell lung cancers than in normal lung, which correlated with the advanced stages of the disease, the grade, and a poorer prognosis (22). A similar effect was observed in renal cell carcinoma in the 2013 study by Teng et al. (23). Migita et al. revealed that ACLY depletion suppressed growth and induced apoptosis in a subset of cancer cell lines including prostate cancer, breast cancer, and colorectal cancer cell lines (24). Wang et al. reported that low ACLY expression was associated with improved overall survival in patients with acute myeloid leukemia (AML), and knockdown of ACLY in THP-1 and MOLM-13 leukemia cell lines caused proliferation arrest (25).

A previous study of ours revealed higher mRNA expression of ACLY in ovarian cancer tissue than in normal ovarian epithelium, and this higher expression predicted poorer prognosis with shorter overall survival (14). Furthermore, we analyzed GEO datasets and TCGA datasets, and obtained similar results.

Most research has focused on the factors influencing the modulation of ACLY. SREBP-1 transcriptionally regulates ACLY expression (26). AKT directly activates ACLY by phosphorylation (22). Acetylation of three lysine residues in ACLY stabilizes the protein by competitively inhibiting ubiquitylation, hence preventing degradation (27).

Only a handful of studies have investigated the mechanisms of ACLY knockdown in malignant tumors. Hanai et al. demonstrated that knockdown of ACLY attenuates PI3K–AKT pathways, and combination with a statin achieves a dual inhibition of lung cancer growth (28). Migita et al. have demonstrated that ACLY inhibition can induce ROS production via the AMPK pathway (29).

Even fewer studies have investigated the connection between ACLY knockdown and resistance to chemotherapeutic drugs. ACLY knockdown is reported to re-sensitize SN38-resistant colorectal cancer cells (30). Targeting ACLY sensitizes castration-resistant prostate cancer cells to androgen receptor (AR) antagonism by an ACLY–AMPK–AR feedback pathway (31). Zhang et al. reported that exogenous addition of citrate to pleural mesothelioma cells induces apoptosis and acts to synergistic effect with cisplatin (32).

In the present study, we found that ACLY knockdown inhibited cell proliferation and induced apoptosis in three ovarian cancer cell lines. In ACLY-knockdown cells, the cell cycle was arrested in the G1 phase, with decreased expression of proteins related to the G1 phase cell-cycle checkpoint, including CCND1 and CDK4. Their upstream regulator, P16, was upregulated in ACLY-knockdown cells. P16 is a canonical negative regulator of CDK4 (33). Endogenous depletion of ACLY has been found to be related to decreased of p-AKT in lung cancer cells (28), in line with our results. To further verify the anticancer effect of ACLY inhibition, we performed xenograft assays in nude mice, which yielded results consistent with our *in vitro* experiments. Our study revealed that knockdown of ACLY inhibited cell proliferation *in vitro* and *in vivo*, probably by modulation of the P16–CCND1–CDK4 pathway and inhibition of p-AKT activity.

In addition, we observed higher levels of P53 expression in A2780 and HEYcells, but not in SKOV3 cells. SKOV3 cells is characterized by homozygous deletion of p53 gene, with deficiency in P53 mRNA transcription. It is well-known that P53 has a vital antitumor role and that P53 activation inhibits the cell cycle and promotes apoptosis (34). Coincidently, we found that the increase of apoptosis due to ACLY knockdown was less apparent for SKOV3 cells than for A2780 and HEY cells. Although P53 mutations have been shown as frequent events in ovarian cancer, wild-type P53 protein was also expressed in cells which embraced recessive P53-mutante allele.

AKT, also known as protein kinase B, is a canonical downstream effector of PI3K (35, 36). The PI3K–AKT pathway has been well-studied as a molecular escape route for cells to avoid death (37). In the context of resistance to chemotherapeutic drugs, it is well-known that activation of the PI3K–AKT pathway not only occurs but also has an important role in multidrug resistance (38). According to Yang et al., AKT promotes resistance to cisplatin in ovarian cancer cell lines by modulating P53 on the caspase-dependent mitochondrial death pathway (39). Clark et al. have reported that AKT activation promotes breast cancer cell survival and therapeutic resistance, and induction of AKT by trastuzumab or tamoxifen treatment reduced the apoptosis induced by doxorubicin (40). Knuefermann et al. reported that HER2–PI3K–AKT activation leads to multidrug resistance in human breast adenocarcinoma cells (41). In addition, Perez-Tenorio et al. reported that activation of AKT is related to resistance to endocrine therapy (42).

Since we showed an active role for AKT in resistance to cancer chemotherapy, and that inhibition of ACLY downregulates the PI3K–AKT pathway, we then wondered if knockdown of ACLY could re-sensitize A2780/CDDP cells to cisplatin. We hypothesized that knockdown of ACLY might decrease the IC_50_ value of cisplatin in A2780/CDDP cells, which was confirmed by the results of MTT assays. We performed cell apoptosis assays on A2780/CDDP-NC and A2780/CDDP-shACLY cells in dose- and time-dependent experiments, and found that A2780/CDDP-shACLY cells responded more sensitively to cisplatin. We also performed *in vivo* tumor formation assays, in which a combination of ACLY knockdown and cisplatin treatment achieved the best curative effect. These collective results verify that the knockdown of ACLY can reduce cisplatin resistance in ovarian cancer *in vitro* and *in vivo*.

With the knockdown of ACLY in A2780/CDDP cells, the expression PI3K, AKT, and p-AKT was lower. Thus, we hypothesized that ACLY acts along PI3K/AKT pathways. We performed a rescue experiment involving AKT overexpression verified this hypothesis. We overexpressed AKT in A2780/CDDP-NC cells and found that IC_50_ value of cisplatin wasn't elevated significantly, and when overexpressed AKT in A2780/CDDP-shACLY cells, IC_50_ showed an obvious elevation. It is then verified that inhibition of ACLY resensitized A2780/CDDP cells to cisplatin through inhibiting AKT.

The AMPK pathway has a key role in metabolic reprogramming, which is also critical in multidrug resistance in cancer therapy. The anti-diabetic drug metformin, also well-known as an AMPK activator, can increase cancer chemosensitivity *in vitro* and *in vivo* (43). ACLY can directly interact with AMPK to inhibit the catalytic subunit of AMPK (44), and Migita et al. have demonstrated that ACLY inhibition might induce ROS production via the AMPK pathway (29). In our study, knockdown of ACLY activated AMPK by increasing levels of p-AMPK-α. Furthermore, ROS levels were elevated in A2780/CDDP ACLY-knockdown cells. As previously mentioned, cisplatin is toxic by production of excess ROS to induce apoptosis; the higher levels of ROS produced by the combination of ACLY inhibition and cisplatin treatment might represent a synergetic effect.

Studies have shown that SB-204990 can effectively reduce the rate of cholesterol and fatty acid synthesis in normal hepatocytes at a concentration of 30 μM, without affecting their proliferation (45). In experimental animal models, SB-204990 was shown to reduce serum cholesterol and fatty acid content, but did not affect the survival time and other physiological indicators of the animal. This inhibitor is currently in clinical use. In diabetic patients, SB-204990 can effectively inhibit platelet aggregation, reduce the occurrence of complications, and improve prognosis (46). Studies on its effect on tumors are still limited to experiments in cells and animals, but it has shown promising antitumor effects in various tumor cell lines and tumor-forming models (47). In the present study, we showed that low-dose SB-204990 did not affect the proliferative activity of A2780/CDDP cells in MTT assays, whereas in conjunction with cisplatin, it alleviated cisplatin resistance by reducing the IC_50_ value of cisplatin, thus the two compounds work synergistically. The successful use of SB-204990 in diabetic patients make its combination with platinum a possible chemotherapeutic regimen for patients with ovarian cancer.

There are limitations in our study, such as the exclusion of primary cisplatin resistance. Due to heterogeneity of ovarian cancer, it is preferable to use patient-derived tumor xenografts or cells to reflect the status of primary cisplatin resistance and retain the heterogeneity. At present, we lack sufficient data to determine the direct interaction between ACLY and the PI3K–AKT pathway. Future studies should also aim to characterize the inhibition of ACLY under the P53 mutant condition.

## Conclusions

In this article, we reveal a novel characteristic of ACLY in acquired cisplatin resistance. We investigated the upregulation of ACLY in ovarian cancer tissue and its correlation with poor prognosis. Knockdown of ACLY caused cell-cycle arrest by activating P16, thus inhibiting CDK4 and CCND1 downstream, and caused apoptosis probably by inhibiting p-AKT. We found that knockdown of ACLY reduced acquired resistance to cisplatin by inhibiting the PI3K–AKT pathway and activating the AMPK–ROS pathway. We discovered that SB-204990, a specific inhibitor of ACLY, might be clinically useful in ovarian cancer patients. However, further studies are needed on the heterogeneity of patient-derived tumors.

## Data Availability Statement

The original contributions presented in the study are included in the article, further inquiries can be directed to the corresponding authors.

## Ethics Statement

The studies involving human participants were reviewed and approved by Ethics Committee of Shandong University (Approval number: KYLL-2019-261). The patients/participants provided their written informed consent to participate in this study. The animal study was reviewed and approved by Experimental Animal Ethics Committee of Qilu Hospital of Shandong University (Approval number: DWLL-2019-011).

## Author Contributions

XW, YW, and PL designed the research process. XW, YW, and WL performed the bioinformatic analysis. JS and YP performed the experiments. XM and QL analyzed the data. XW, YP, and RL wrote the paper. All authors read and approved the final manuscript.

## Conflict of Interest

The authors declare that the research was conducted in the absence of any commercial or financial relationships that could be construed as a potential conflict of interest.
