# Corrigendum: Targeting ACLY Attenuates Tumor Growth and Acquired Cisplatin Resistance in Ovarian Cancer by Inhibiting the PI3K–AKT Pathway and Activating the AMPK–ROS Pathway

**DOI:** 10.3389/fonc.2021.742374

**Published:** 2021-09-07

**Authors:** Xuan Wei, Juanjuan Shi, Qianhan Lin, Xiaoxue Ma, Yingxin Pang, Hongluan Mao, Rui Li, Wei Lu, Yu Wang, Peishu Liu

**Affiliations:** ^1^Department of Obstetrics and Gynecology, Qilu Hospital of Shandong University, Jinan, China; ^2^Key Laboratory of Gynecology Oncology of Shandong Province, Qilu Hospital of Shandong University, Jinan, China; ^3^Shandong Engineering Laboratory for Urogynecology, Qilu Hospital of Shandong University, Jinan, China; ^4^Department of Gynecology and Obstetrics, Affiliated Tengzhou Center People’s Hospital of Jining Medical University, Tengzhou, China

**Keywords:** ACLY, ovarian cancer, cisplatin resistance, PI3K-AKT pathway, AMPK-ROS pathway

## 

In the original article, there was a mistake in **Figure 5B** as published. We recognized by ourself that the picture of “PI3K” was the same as that of “pan-AKT”, we made a mistake when we dragged the original figure into the AI software.

The corrected **Figure 5B** appears below.

**Figure 5:**
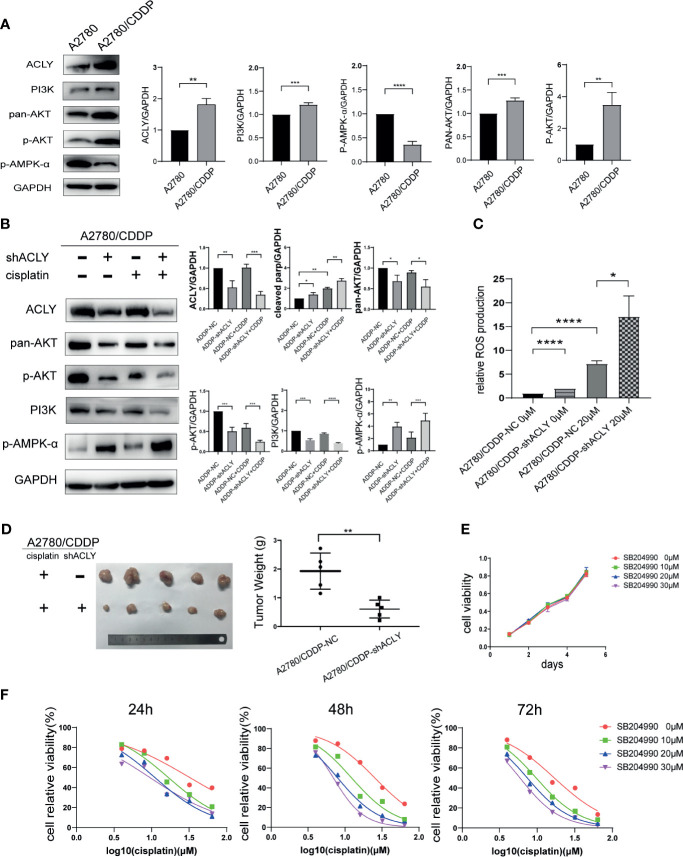
ACLY knockdown inhibited PI3K/AKT pathway and activated AMPK pathway. **(A)** Western blotting was used to detect the differential expression of ACLY, PI3K/AKT pathway and p-AMPK-a in A2780 and A2780/CDDP cells. **(B)** Western blotting on A2780/CDDP-NC and A2780/CDDP-shACLY cells, and them under 20μM cisplatin treatment for 48 h, the bands were quantified and analyzed. The bands were quantitated with Image J software, statistical analysis was performed using Student’s t-test. **(C)** ROS production of the aforementioned cells and them under treatment of 20μM cisplatin for 48 h, statistical analysis was performed using Student’s t-test. **(D)** Tumor xenograft formation of A2780/CDDP-NC and A2780/CDDP-shACLY cells with treatment of cisplatin, with each group containing five mice. The difference in tumor weights was compared using Student’s t-test. **(E)** Proliferation of A2780/CDDP cells in respond to different concentration (low-dose, 10–30μM) of SB-204990, the growth curves were analyzed using one-way ANOVA test. **(F)** 24, 48, and 72 h IC50 of A2780/CDDP cells under treatment of cisplatin combined with different concentration of SB-204990 (from 0 to 30μM), 24 h IC50 of which were 32.34 (26.71–40.60), 16.75 (15.24–18.43), 11.08 (9.736–12.55), 9.495 (7.759–11.38) μM, respectively; 48 h IC50 of which were 25.37 (23.86–27.00), 12.33 (10.74–14.13), 7.983 (7.487–8.499), 6.979 (6.749–7.215) μM; 72 h IC50 of which were 16.96 (14.89–19.34), 9.727 (9.294–10.18), 7.407 (7.083–7.741), 5.922 (5.601–6.246) μM, respectively. All cell experiments were repeated three times at least. *P < 0.05, **P < 0.01, ***P < 0.001, and ****P < 0.0001 for statistical analysis of the indicated groups.

The authors apologize for this error and state that this does not change the scientific conclusions of the article in any way.

## Publisher’s Note

All claims expressed in this article are solely those of the authors and do not necessarily represent those of their affiliated organizations, or those of the publisher, the editors and the reviewers. Any product that may be evaluated in this article, or claim that may be made by its manufacturer, is not guaranteed or endorsed by the publisher.

